# The managerial role of healthcare professionals in public hospitals: a time-driven analysis of their activities

**DOI:** 10.1186/s12913-023-09395-7

**Published:** 2023-05-10

**Authors:** Lorenzo Pratici, Andrea Francesconi, Gianluca Lanza, Antonello Zangrandi, Simone Fanelli

**Affiliations:** 1 Department of Economics and Management, University of Parma, Via J. F. Kennedy, 6 - Parma (PR), Parma, Italy; 2 Department of Economics and Management, University of Trento, Trento, Italy

**Keywords:** Clinician-manager, Time-driven analysis, Health services management, Public hospitals

## Abstract

**Background:**

New Public Management theory affected reforms of public sectors worldwide. In Italy, an important reform of the healthcare sector changed the profile of public hospitals, creating new management related positions in 1992. The reform defined the role of the clinician-manager: a hybrid figure, in charge of managing an entire unit. This paper aims to investigate how much clinician-managers feel like managers and how much they still feel like professionals, using time as a driver to conduct the analysis.

**Methods:**

A survey-questionnaire was administered to a set of 2,011 clinician-managers employed in public hospitals, with a response rate of 60.42%. The managerial role of healthcare professionals in public hospitals: A time-driven analysis of their activities. The questionnaire aimed to identify the difference between how much time clinician-managers actually spend on daily activities and how much time they would think be appropriate. To better cluster different type of management styles, subgroups were identified based on the type of organisations respondents work for, geographical location, and professional specialty.

**Results:**

Findings suggest that clinician-managers spend more time on clinical activities than management. Clear differences are found according to professional specialty, and there are fewer differences in geographical location and the type of organisation.

**Conclusions:**

The absence of clear differences in the responses between different geographical areas implies that a shared organisational culture characterizes the whole sector. However, differences in how the clinician-manager role is perceived based on the professional specialty suggest that closer integration may be needed.

**Supplementary Information:**

The online version contains supplementary material available at 10.1186/s12913-023-09395-7.

## Background

In the 1980 and 1990 s, the spread of New Public Management (NPM) theory brought important changes in the functioning of public administration, and reduced many of the inefficiencies typical of the bureaucratic model. In theory, NPM enabled the shift from procedure-based to result-based administration, with effects on its efficiency and efficacy. However, reality does not always reflect theory.

Hood (1991) identifies several conceptual elements of NPM, including recognition of the importance of professional management, the need for performance evaluation tools; the focus on results; and the implementation of managerial logic at all levels of responsibility [1]. The introduction of managerial logic is widely considered necessary to ensure the sustainability of the public sector through the decentralization of power, greater managerial autonomy, the introduction of control systems and performance evaluation and the spread of managerial culture [2].

Healthcare was the first public sector to be subject to NPM. The need to increase attention to costs and the levels of efficiency and effectiveness of healthcare organisations has prompted many countries around the world to start reform processes in healthcare systems inspired by the managerial logic typical of the private sector. These reform processes have very often also affected healthcare professionals, asking them to develop a certain focus on the organizational and management methods of resources in clinical processes.

There are also issues in the NPM approach to professionals [3]. NPM has in fact an ambiguous attitude towards expertise and professionals. On one hand, professionals are the key to better performance. The NPM concept “Let managers manage” reflects confidence in the professionalism of managers [4], who are expected to act as leaders empowering staff to do their best [5]. However, NPM, and particularly performance management systems, often express a certain amount of distrust in professionals [6].

At the same time there is a strong belief that physicians in leadership and management roles contribute to a more efficient and higher quality system [7]. Physicians today are required to have an understanding of and some training in professional management skills. Where both clinical and professional leadership is combined in one role, patient care and outcomes are improved [8]. Physicians trained as managers are thus a hybrid role for a hybrid organisation, and have a positive impact on their patients while also contributing to the improvement of their organisation and, as such, the entire system.

For all these reasons, reforms have often aspired to producing the clinician-manager^1^, a hybrid professional performing clinical and management duties.

### The clinician-manager: a hybrid role

The need for a more “management-oriented” organisation of the healthcare sector is given by several endogenous factors which are not under the control of any individual organisation [8, 9]. These includes cutbacks in resources, technological innovation requiring increasingly costly investments, rising demand for quality healthcare, and reforms in many OECD countries which have altered administration and financing of healthcare systems [10].

The hybrid figure of clinician-manager thus appeared in all developed countries, and has become one of the key issues in healthcare systems. The United Kingdom, for example, following the Griffiths Report of the late 1980s, gave hospital doctors responsibilities for management alongside clinical duties [11]. Denmark did the same in the 1980s, moving to new organisational models based on the hybrid role of the clinician-manager [10]. Other countries like France and Spain took the same path in the 1990s and early 2000s [12]. For Italy, the turning point was the 1992 Health Reform, which established health organisations as independent entities, and stressed the need for careful costing, management and efficiency.

There has been much research, and there is a wide area of literature looking at how the clinician-manager can or should combine technical professionalism with managerial efficiency. Clinicians-managers have to balance and mediate between the two different worlds, managerial and professional. Each follows different and often conflicting lines of logic [13]. Some authors suggest that clinicians can be efficient managers thanks to their professional role [10], but others disagree [14].

Mintzberg [15] applied an empirical inductive approach to studying how managers use their time. The Mintzberg’s inspired a stream of further studies that used the method of structured observation in various managerial occupations [16–18]. Thus, today, several scholars find that the most useful driver for measuring the level of activity in any type of organisation is time [19–21].

### Managers vs. clinicians: a time-driven analysis of clinician-manager activities

Time can be considered as the only economic resource common to all managers, regardless of the organisation they work in [22] and temporality is central in measuring how much work managers accomplish for a given task or effort [23]. But despite wide agreement that it is a useful variable to take into consideration, it is rarely used as a driver in management [23, 24].

This paper aims to examine the role of clinician-managers using a time-driven analysis, considering actual time use, or how much time managers actually spend on certain activities, and desirable time use, or how much time they believe should be allocated to the same activities.

#### Actual time perspective

Although many studies on managers’ use of time concern actual time, the area is relatively unexplored and there are few published studies [23, 25]. Direct examinations of managerial time use and task accomplishments include structured diaries recording activities, observation and activity sampling. However, it is not always easy to measure time really spent on something, and this has long been recognized as a problem. Braithwaite and Westbrook (2011) conclude in fact that indirect methods, such as questionnaires and interviews seeking time estimates, are also effective [23]. Direct methods, although they yield more reliable data, are typically more time-consuming and difficult to practice. Indirect methods are thus considered as a useful tool, a proxy for reality because, despite their obvious disadvantages, they are based on cognitive assessments of tasks as opposed to everyday physical actions. Furthermore, the bias arising from inaccuracy in time perception can be limited by extending the analysis to a large sample of respondents or interviewees [26].

#### Ideal time perspective

The ideal time dimension provides a picture of clinician-managers wishes about how they should use their time, under ideal conditions. It exploits their on-field experience, and compared to the real time perspective, may produce useful insights on what should be re-taken into consideration for policy makers [23]. Managerial work is found to be characterized not by the orderly carrying out of predefined and prescribed functions but by discontinuity, interruption and often knee-jerk reaction to presenting issues and problems [25]. This implies that much of the clinician-managers day does not proceed as they plan, or as they would consider appropriate. There is very little research on the actual time perspective and even less on ideal time. The honourable exception is the study by Braithwaite and Westbrook (2011), which first made a comparison between actual and ideal time perspectives [23].

### The figure of clinician-manager in the italian NHS

In Italian healthcare, the role of clinician-manager, representing the middle management of health organisations, was created by the 1992 reform (Legislative Decree nr. 502/1992). The reform reorganised the structure of public hospitals along lines of managerial accountability in a clear drive for financial, technical, and functional efficiency [22]. Among other things, the reform transformed the role of the clinician who assumed responsibility for the organizational unit, requiring him/her to become manager [10]. As matter of fact, the new chief of unit is still a clinician, who is also responsible for running and organising the structure, planning and scheduling projects, managing clinical outcomes, managing human resources, and overseeing financial, technical, and administrative targets [27].

As in many other countries, this was an epoch-making change, in compliance with what NPM currents were anticipating, and required the integration of the professional skills of clinicians with managerial skills unrelated to their traditional training [28].

Management training programs were put in place across the country to encourage and support the change in the role and responsibilities of clinicians. Over time, these programs have become mandatory for physicians holding positions of responsibility in Italian healthcare organisations [10]. It was hoped that the gap between clinical culture and managerial culture would be bridged. The programs have not however always been successful in overcoming strong resistance to the shift from a professional to a clinical governance-oriented culture [29]. Resistance was (and still today is) often related to the difficult process of changing the role of clinicians. As a matter of fact, a common misconception often brought up sees the clinician abandoning the role of “a proper doctor” to become a mere administrator. This is not only completely inaccurate, but also basically the opposite of the *ratio legis* as intended by the Legislator.

Howeve, despite government intention is clear, the same cannot be said about how to measure the extent to which an individual should be a manager and to what extent a clinician. Measuring and assessing the work of a physician can be a tricky and it is hard to identify a driver that can be applied universally [30] and, as such, this paper addresses this issue.

### Proposition development

Generalization can often be misleading, so here clustering is performed in three dimensions: size, type and geographical region of units, as described below.

#### Differences in activities: clinical vs. managerial duties

A key issue in healthcare reform has been the medical profession’s reluctance to adopt management values [31]. Clinicians are trained along narrow professional lines which often take no account of the wider inter-professional and organisational factors within their employing organisations [32]. Very often managerial priorities (such as cost control, accountability, teamwork) are considered by clinicians as limitations to their work activities and their levels of autonomy, and they consider clinical competence as the only source of legitimacy of their actions [33].

To this is added that previous studies have shown that clinician-managers nationwide often feel unprepared for their management duties,and that clinician-managers feel more comfortable in performing their professional duties than in acting as real managers [10, 29].

Nonetheless, a 2009 survey published by Lega and Prenestini [34] found that the great majority of Italian doctors aim to become hospital managers in their career, so this finding may seem contradictory.

This leads to the first proposition (P1).

P1 – *clinician-managers aim to spend more time on clinical duties than on management*

#### Size differences: small units vs. large units

Like any organisation, healthcare organisation management needs to identify the best management approach for each unit [35]. No single model fits all, as every organisation has its own particular features. Variables like staff composition, geographical location and size all impact on the choice of strategy [36].

The size of a unit is a crucial issue for policy makers [37]. Larger units are more complex to manage, and require procedures for the integration of all staff [38]. Routine activities, procedures, and checklists are widely used in larger units [39]. However, this can bring a lack of flexibility so it is important to define some sort of autonomy for middle-management [35, 40]. The balance between flexibility and procedural approach is thus a major issue in any type of large units. The implementation of a strong coordination system is found to be the main effective solution [41]. Healthcare organisations are characterized by a high level of specialization, and are “organisational silos” as theorised by Pratici et al. [8]. Coordination mechanisms are the responsibility of middle management, so it may be expected that in larger units, professionals pay more attention to management than to clinical duties. This syllogism, applied to hospital units, leads to our first proposition (P1):

P2 – *Clinician-managers in larger units tend to spend more time on management compared to clinician-managers in smaller units.*

#### Differences between clinical specialties: surgery units vs. medical units

The professional perception of clinicians at any level has a huge influence on their roles in organisations. Clinicians tend to be oriented towards the development of their technical skills in order to be recognized as a leader [42, 43]. In medical communities, there appears to be a difference between the two categories of surgeons and medical professionals, according to the perception of the importance of the managerial skills required by their role [44]. Surgeons are more oriented to recognizing the value of their technical skills, rather than their managerial skills, and they perceive technical skills as more important for career development [45]. This yields the second proposition:

P3 – *surgeons tend to spend much more time on clinical duties than other clinician-managers*

#### Regional differences: North vs. Centre vs. South

In Italy, although the health service is national, responsibility for hospital management is held by Regional Authorities. Regions can thus implement organisational and managerial models that best suit their needs, but they leave the task of defining the best management approach to single healthcare organisations [10]. For this reason, inter-regional differences may create disparities in the role of clinician-managers. Furthermore, despite the existence of national policies, these are not necessarily applied evenly by all regional authorities, which increases the risk of disparity in the quality of care provided across the country [46]. Rather, several studies have demonstrated different organizational models between Italian Regions and different performances, with better results in the Northern Regions than in the Southern ones [43, 46].

It can therefore be expected that regional policies strongly influence hospital management approaches, leading to the fourth proposition (P4).

*P4 – Given the disparities between regional healthcare systems, there is a clear difference in the role of the clinician-manager in different regions.*

The four propositions are tested following the methodology described in the next section.

## Methods

This research uses a two-phase quali-quantitative analysis, based on focus group discussion and a survey questionnaire.

Phase one designed the content of the questionnaire and was conducted through a focus group consisting of health management experts. “Clinician-manager activities” were identified, so that time actually spent and desired amount of time could be calculated.

Focus group members were selected among management scholars and needed to satisfy two criteria: (1) being a scholar in health management issues and (2) having published at least one paper in the last five years in a peer-reviewed Scopus indexed journal.

The focus group met for two separated sessions and identified a total of six overall activities as follows:

- Clinical activity (e.g., patients care, clinical records, patients’ relations, etc.).
- Management activities (e.g., auditing, definition of care pathways, guidelines, personnel evaluation, etc.)
- Non-clinical activity (e.g., emails, formal procedures, shift planning, etc.)
- Internal relations within the organisation (e.g., formal or informal meetings, fellows’ relations, etc.)
- External relations with stakeholders (e.g., budgeting, trade union relations, committees, providers, other organizations, etc.)
- Education and research activities.

These six activities were used as items in the questionnaire (Phase 2) (see Additional file), which was administered to middle management in hospitals equally distributed throughout Italy. The study relates to questionnaires filled in between March 2021 and September 2021.

The validity and effectiveness of the questionnaire were confirmed by a pilot test on 47 respondents: 20 of which meeting all the requirements for membership of the focus group (management scholars) and 27 being clinician managers.

Once tested, all 6,115 clinician-manager working in public Italian hospitals have been emailed asking to participate to the research. Only 2,011, corresponding to 38.27% of the whole set of Italian clinician-managers, have actually declared themselves interested in taking part to the research. However, the final number of respondes obtained consisted in 1,215, with a response rate of 60.42%. Out of the total 1,215 responses collected, 203 were excluded as considered non-usable (e.g. they didn’t fill in the biographical part of the questionnaire). This makes the final sample composed by 1,012 responses. Appendix 1 shows the sample characteristics.

The questionnaire consists of two parts. The first identifies the general characteristics of the respondent: his/her organisation, age, unit, the region in which he/she works, the organisation currently employing him/her. The second part consists of two overall questions, each with a panel of 6 items, the set of activities identified above. Question 1 (Q1) asks “*If 100 is your total professional time, how much time do you spend on each of the following activities?*”. Respondents indicate a number *n*, representing time spent, such that 0 < *n <* 100.

Question 2 (Q2) asks “*How do you assess the time you spend on each activity?”* and is based on a semantic-dimensional three point scale, but represented as a Likert scale of 1 to 5, where 1 is “insufficient”, 3 is “adequate”, and 5 is “excessive”.

This scale made it possible to measure whether respondents considered the time they spend on each activity to be adequate. The main purpose of the analysis is indeed to correlate the actual time spent on activities with the assessment of health professionals on the adequacy or appropriacy of such time. To make the scales comparable, Q1 was standardized to a *5* point scale. Following Hofstee et al., (1998), standardisation was made in percentiles. If the respondent lies between the first and the 20th percentile, the score is 1; if the respondent lies between the 21st and the 40th percentile, the score is 2; etc. [37]. Table 1 shows the conversion algorithm.

**Table 1:** Standardisation conversion

Percentile	Score assigned
1–20	1
21–40	2
41–60	3
61–80	4
81–100	5

Responses were analysed using STATA®, software version 14.1, and the internal consistency of the questionnaire was calculated. All items reported a score > 0.7. Results are described in the section below. First, scores for the whole sample are reported, and items are ranked. Independent t-tests and one-way analyses of variance were then performed to compare the level of time spent on each item to the assessment of the adequacy of this level of time. Subgroups were identified from information provided in the first part of the questionnaire. They are based on the type of organisation respondents work for, geographical location, and professional specialty. Because medians did not correspond to means, the asymmetry index was calculated to test the hypothesis of normality. All analyses yielded were carried for alpha equal to 0.1. The deviations between responses to Q1 and Q2 were calculated, this deviation being the difference between desirable time and actual time spent on each of the six activities. T-tests were also run to provide statistical significance for results.

### Ethical approval

The present study was not submitted to an institutional ethics committee since this is not required under Italian legislation. All survey respondents gave their written consent to participate after being informed about the study.

All experiments and analyses were performed in accordance with relevant guidelines and regulation (e.g. Declaration of Helsinki, available at the following link: https://www.wma.net/what-we-do/medical-ethics/declaration-of-helsinki/).

All survey respondents gave their written informed consent to participate after being informed about the study.

## Results

This section outlines results for the three sub-groups.

### Real time vs. desirable time

Table 2 analyses the amount of time spent by clinician-managers on each of the six activities identified by the focus group. The first two columns report the time actually spent by clinician-managers on each activity, and the last two columns report the time they consider desirable or ideal. The “Time dedicated” section reports standardised means as well as real means. The “Desirable time” reports means of the Likert scores, as well as the distance from the “Adequate” level, which is 3 on a scale of 5. Where the deviation from the adequate level shows a negative value, this shows that respondents feel they spend too much time on an activity. Where the deviation shows a positive value, respondents feel they spend too little time on that activity. The closer the deviation is to zero, the more respondents feel that the time spent is adequate.

**Table 2:** Actual time vs. desirable time

*Activity*	Actual time		Desirable time	
	**Standardized Mean**	**Actual Mean**	**Desirable time**	**Distance from adequacy**
*Clinical activity*	3.19	31.79	2.39	0.61
*Management activity*	3.65	16.93	2.55	0.45
*Non-clinical activity*	3.43	17.80	4.11	-1.11
*Internal relations*	3.35	14.13	2.65	0.35
*External relations*	3.35	10.54	3.17	-0.17
*Education & research*	3.45	9.86	1.66	1.34

All the areas are identified scores lie in the 3rd percentile (60%) but with a high level of variance, as measured by standard deviation. Variance, however, is much higher in the Actual time than in the Desirable time section.

In the Actual time section, all activities lie in the 3rd percentile, although there are some major differences. Clinical activity for example is much closer to the 2nd percentile, while management activities are much closer to the 4th percentile. Non-standardised means reveal a general prevalence of time spent on clinical activities; an average of 31.79% time is spent on these. Non-clinical activities come far behind, and is ranked second, but with standardized measures ranked third. Far too much time appears to be spent on Non-clinical activities (4.11), while very little time is spent on Education and research. All other activities fall at a distance < 1 from adequacy. Independent T-tests and ANOVA were then run to test potential differences between internal groups of respondents.

The results do not confirm P4; time is considered insufficient for both clinical and management activities. There is a 0.61 distance for adequacy for clinical and 0.45 distance for management activities.

### Size differences: small units vs. large units

Table 3 reports single scores in the actual time and desirable time sections for the two subgroups: those in smaller units (20 or fewer persons) and those in larger units (21 or more persons). The T-test showed deviations in variables between subgroups statistically different from zero (alpha < 0.1).

**Table 3:** Comparison between large and small units

Activity	Actual time					Desirable time				
	<= 20	> 20	Diff. between means	F	P	<= 20	> 20	Diff. between means	F	P
	Mean	Mean				Mean	Mean			
*Clinical activity*	2.96	2.68	0.28	3.54	0.00*	2.51	2.21	0.30	3.12	0.93
*Management activity*	3.63	3.89	-0.26	1.98	0.87	2.54	2.68	-0.14	2.17	0.53
*Non-clinical activity*	3.38	3.70	-0.31	1.77	0.05*	3.36	4.07	-0.71	4.30	0.00*
*Internal relations*	3.41	3.86	-0.45	2.02	0.05*	1.89	2.61	-0.71	3.91	0.45
*External relations*	3.37	3.64	-0.27	2.84	0.36	3.02	3.05	-0.03	1.98	0.06*
*Education & research*	3.46	3.27	0.19	4.17	0.81	1.71	1.49	0.22	3.02	0.14

* Alpha < 0.1

The biggest deviation in the Actual time section occurs in Internal relations (–0.45), followed by non-clinical activities (–0.31). More time is spent on these activities in larger units. In general, activities relating to a more managerial role (Management activities, Non-clinical activities, Internal and External relations) seem to be more time-consuming in larger units, while more time is spent on Clinical activities and Research and education in smaller units (differences + 0.28 and + 0.19).

Desirable times fall into the same clusters for both large and small units, above and below the adequacy time set to 3 on a scale of 5. However, variance between scores appear to be larger than in the Actual time section. A deviation of − 0.71 is registered for both Non-clinical activities and Internal relations, which is lower in smaller units, showing that respondents consider the time spent on Non-clinical activities and Internal relations is more adequate than in larger units.

However, scores for Internal relations appear to be not statistically significant, with p > 0.1, so P1 appears to be confirmed; much more time is spent on management and consistently less on clinical duties in larger units.

### Clinical specialty differences: surgery units vs. medical units

Table 4 shows single scores in Actual time and Desirable time sections for the two subgroups surgeons and other medical specialties. The T-test shows deviations in variables between subgroups which are statistically different from zero (alpha < 0.1).

**Table 4:** Comparison between surgeons and other clinicians

Sets of activities	Real time					Desirable time				
	Surgeons	Other areas	Diff. between means	F	P	Surgeons	Other areas	Diff. between means	F	P
	Mean	Mean				Mean	Mean			
*Clinical activity*	4.23	2.89	-1.34	2.78	0.00*	2.61	2.42	-0.19	3.37	0.01*
*Management activity*	3.58	3.88	0.30	1.77	0.00*	2.54	2.68	0.14	2.02	0.00*
*Non-clinical activity*	2.85	3.59	0.74	1.24	0.00*	3.82	3.95	0.13	2.13	0.11
*Internal relations*	3.21	3.86	0.65	1.98	0.05*	2.73	2.24	-0.49	3.18	0.00*
*External relations*	3.51	3.54	0.03	2.01	0.00*	3.11	2.99	-0.12	1.72	0.00*
*Education & research*	3.47	3.43	-0.04	3.12	0.00*	1.78	1.65	-0.13	1.74	0.00*

* Alpha < 0.1

These subgroups present the most differences. Surgeons tend to spend a consistently higher amount of time on clinical activities than management in surgical departments (4.23) than in others (2.89). However, Desirable time is assessed to be almost the same (2.61 vs. 2.42, in both cases < 3 with a difference of – 0.19). The deviation between actual time spent and desirable time is much larger than for other activities. Surgeons spend much less time than other clinicians on the second activity, non-clinical work, with a difference between means of + 0.74. Again, Desirable time seems not to differ much, and in this case the difference is not statistically significant.

Other scores appear to be substantially similar in both groups. This confirms P2: surgeons tend to spend much more time dealing with clinical duties than with managerial activities, if compared to other medical specialties.

### Regional differences: North vs. Centre vs. South

Table 5 reports regional differences between 3 macro-regions: North, Centre and South as classified by ISTAT (Italian Institute of Statistics). The first part shows Actual time scores, and the second part Desirable time scores. Analysis of variance is conducted to highlight potential differences between means.

**Table 5:** Comparison between macro-regions

Activity	North			Centre			South		
	Mean	F	P	Mean	F	P	Mean	F	P
*Clinical activity*	3.16	3.54	0.00*	3.19	2.58	0.00*	3.27	3.37	0.01*
*Management activity*	3.55	1.98	0.00 *	3.85	1.17	0.00*	3.82	2.23	0.00*
*Non-clinical activity*	3.46	1.77	0.06*	3.40	3.13	0.00*	3.32	2.21	0.00*
*Internal relations*	3.45	2.02	0.11	3.32	1.19	0.02*	3.04	1.99	0.07*
*External relations*	3.34	2.84	0.10	3.40	1.12	0.02*	3.32	1.13	0.15
*Education & research*	3.37	4.17	0.01*	3.49	1.11	0.00*	3.65	2.1	0.00*
*Clinical activity*	2.44	3.20	0.00*	2.53	3.13	0.021*	2.45	2.98	0.17
*Management activity*	2.58	2.30	0.00*	2.46	1.23	0.00*	2.49	1.18	0.19
*Non-clinical activity*	4.01	2.10	0.05*	3.77	1.22	0.00*	3.79	1.78	0.00*
*Internal relations*	2.66	1.10	0.13	2.52	1.43	0.00*	2.39	1.19	0.01*
*External relations*	3.10	1.19	0.17	3.02	1.54	0.01*	2.79	2.01	0.19
*Education & research*	1.59	1.17	0.22	1.71	1.19	0.13	1.75	1.73	0.22

* Alpha < 0.1

Analysis of the scores reveals very little difference between the three macro-regions. All rankings are the same and all scores fall into the same percentile for each activity. There does however appear to be a slight tendency to spend more time on management than Clinical activities and Education and research in the North. Furthermore, in northern regions, scores calculated for the Desirable time tend to be slightly further from the adequacy level. In other words, they lie at a greater distance from 3 than in the Centre and South.

These results do not confirm P3; there is no difference or very little difference, which is statistically insignificant, between different regions.

## Discussion

This study aimed to estimate how much time clinician-managers allocate to management compared to clinical duties, as well as the perception of the adequacy and appropriacy of this time among clinician-managers.

Health professionals, in general, are increasingly required to combine managerial activities with their clinical activities. This phenomenon affects most of the health system of industrialized countries around the world, and although the various health systems differ in their main structures as well as available resources [10], it is widely recognized that nowadays professionals need to focus their attention on both clinical and management aspects of their work [47].

The 1992 reform in Italy created a hybrid figure and changed the management of public health organisations radically [48]. In the 1990s, there was widespread unpreparedness for management duties among clinician-managers in many countries [49], but central government often provided training and schemes for improving management culture in public hospitals [50]. Despite these efforts, however, other research finds that clinician-managers still feel somewhat unprepared for management duties [9, 10]. It thus appears crucial to investigate the current state of art, and discover, in other words, whether clinician-managers feel themselves to be managers: if clinician-managers feel unprepared for managerial duties, they may dedicate less time such activities. However, it could also be that since they dedicate not as much time as they should, this may lead them to feel less prepared.

As noted above, there is no single model fitting all contexts, so for this research, several subgroups were created to investigate variability, and four propositions constructed. The results corresponding to each proposition are discussed below.

*P1 (Not confirmed) – Clinician-managers aim to spend more time on clinical duties than management.*

Studies on clinicians becoming managers are not new in organisational theoretical studies in the healthcare sector, and many have attempted to describe the transition [3, 20, 51]. However, the theory has evolved during the years, as has the hybrid role of clinician-managers. Until the 1980s, the role hardly existed, as clinicians who became administrators usually left clinical duties. At the end of the 1980s, however, NPM theory defined a new hybrid role of a highly specialized professional also performing managerial duties [9].

The perception of how much time should be spent on management and how much on clinical practice has also evolved, and today there is much debate on time allocation. This research attempted to answer to some of these questions, and compared time actually spent with desirable amounts of time for different activities.

A reviewing of the literature confirms how difficult the transition from professional to professional with management duties can be [10]. The 1992 reform in Italy aimed at promoting a new culture among health professionals and enhancing the role of management in public hospitals. Current research however still finds general inconsistency in practice [10, 52].

*P2 (Confirmed) – Clinician-managers in larger units tend to spend more time on management compared to clinician-managers in smaller units.*

There is widespread academic agreement that larger organisations are usually subject to a stronger separation of professional and managerial roles [10, 53]. Looking at hospital units and the hybrid clinician-manager role, it seems that in larger units, clinician-managers tend to carry out more management duties than colleagues working in smaller units. This probably reflects the strong separation of roles, which influences procedures and management culture within the organisation [20, 54]. Our findings are however that there is little difference between larger and smaller units; in both types clinician-managers would prefer to allocate more of their time to clinical duties.

*P3 (Confirmed) – Surgeons tend to spend much more time on clinical duties than other clinician-managers*.

The principle expressed in P3 is not new to the literature either [42, 43], but to the best of our knowledge, no previous empirical analysis has been made in this area. Hypothesis developed by previous scholars appear to be confirmed.

*P4 (Not confirmed) – Given the disparities between regional healthcare systems, there is a clear difference in the role of the clinician-manager in different regions.*

Italy is one of the few countries around the globe to have a National Health Service (NHS) and universal coverage [30]. The Italian NHS is modelled on the British NHS [55], but unlike the British one, the Italian NHS is divided into different regional authorities [30]. Although they are all subject to the same basic legislation and, to some extent share a similar culture [56], several authors suggest that there are big differences in practice [57].

Results from this research however suggest the opposite, and reveal a general shared culture across the country. This is in line with results in other strands of literature. For instance, Calciolari and Ilinka (2016) find that despite the co-existence of different types of organisational culture among health professionals across Italy, they are all guided by the same principles, contributing to create a common ground [30, 58]. Pratici et al. (2022) also find that all public health organisations are devoted to the improvement of the performance of the entire NHS, so the view of clinician-managers is consistent throughout the country [8]. A shared organisational culture is often considered the key to improving the performance of a complex organisation, such as a NHS.

In consideration of all propositions analysed, it is possible to conclude that there are significant differences characterising two clusters of clinical culture: surgeons and other clinicians. Among surgeons, traditional views of the role, and a clear prevalence of clinical over management skills prevail. But among other clinicians, there is evidence of growing awareness of their role as manager.

As for the geographical disparity throughout Italy, clinicians’ personal values tend to be similar, and the decentralisation of decision-making at regional level does not appear to impact on the issues addressed in this paper. As for differences between surgeons and other clinicians, clinical culture appears to be similar even in highly differentiated institutional settings.

Furthermore, management training initiatives to support change have contributed to changing the clinical culture, but even over an extended period of time, have not clearly defined or standardised the managerial role of clinicians. There is the need to maintain investments in training schemes in order to reinforce and support past investments for a continuous and steady alignment between professional and managerial culture.

## Conclusion

This study shows the impact that NPM theory has had on the clinical culture of healthcare organisations, 30 years after the implementation of NPM measures. In general terms, there appears to be a greater awareness of their role among clinicians. This does not however necessarily mean role sharing. The study has important implications for policy choices and future research.

The data provides a complex picture of the relation between clinical managers and their role. It is interesting to note that they have widely accepted managerial duties, but at the same time, many perceive them as simply “bureaucratic stuff”. Today it appears to be positive to leave middle management responsibilities to clinicians rather than staffing healthcare units with new administrators. But even after thirty years, there is still a need for education and support measures to increase the perception of the value of managerial dimensions such as evaluation, leadership, and coordination, etc. as useful rather than too bureaucratic.

The challenge for the system and the different actors is to ensure a balance between the clinical and managerial roles of clinical managers. Given the shortage of resources predicted for the near future, they need to be helped in implementing actions and behaviour, and supported with procedures and tools to improve their management effectiveness.

A further point reached by this research consists in highlighting a relevant issue among Italian public hospitals. Currently, according to the existing organizational model arising from the last reform in 1992, clinical managers are responsible of at least three aspects: quality of services, management of their team, and planning both clinical and non-clinical activities. Analyses performed suggest that clinician-managers are well aware of this role but yet, 30 years after the reform, some resistances are still hampering a full development of this same role.

This opens up further perspectives for future research: should clinician-managers still be in charge of all the activities mentioned in this work? Such a choice would require an institutional change at the national level. Thus, this study aims to provide to national decision-makers several insights of the current situation among Italian public hospitals, sketching a profile of public hospitals middle management, 30 years after the 1992 reform.

This study is however not without limitations. It is known that different work contexts and different institutional settings as well as different social, economic and welfare policy conditions, may very well influence expectations and values of individuals towards the organizations they work for.

Furthermore, generalization in these types of study is hard to be accomplished: the nature of the organization, the regional or national health system in which the organization is working, the epidemiology of the population, and many other variables may affect the validity of the study, if applied to other contexts.

## Electronic supplementary material

Below is the link to the electronic supplementary material.

Supplementary Material 1

Supplementary Material 2

## Data Availability

The datasets during and/or analysed during the current study available from the corresponding author on reasonable request. The questionnaire administered to conduct the analyses constitutes appendix 2 of this paper.

## Footnotes

1 With clinician-managers, we refer to the head of unit (i.e. *Responsabile di Struttura Complessa* in the Italian NHS).

## Abbreviations

NPM: New Public Management
Q1: Question 1
Q2: Question 2
NHS: National Health System

## References

[1] Hood C (1991). A public management for all seasons?. Public Adm.

[2] Wynen J, Verhoest K, Ongaro E, Van Thiel S, Network COBRA (2014). Innovation-oriented culture in the public sector: do managerial autonomy and result control lead to innovation?. Public Manag Rev.

[3] Bellé N, Ongaro E (2014). NPM, administrative reforms and public service motivation: improving the dialogue between research agendas. Int Rev Adm Sci.

[4] Pronovost PJ, Weast B, Holzmueller CG, Rosenstein BJ, Kidwell RP, Haller KB, Feroli ER, Sexton JB, Rubin HR (2003). Evaluation of the culture of safety: survey of clinicians and managers in an academic medical center. BMJ Qual Saf.

[5] Kettl DF (1997). The global revolution in public management: driving themes, missing links. J Policy Anal Manage.

[6] Spreitzer GM, Quinn RE (1996). Empowering middle managers to be transformational leaders. J Appl Behav Sci.

[7] Van Dooren W, Thijs N (2010). Paradoxes of improving performance management (sytems) in public administration. EIPAScope.

[8] Pratici L, Fanelli S, Zangrandi A. Not only funding: how healthcare organizations can contribute to National Health Service sustainability.Int J Public Admin. 2022;1–11.

[9] Lega F, Sartirana M. (2016). Making doctors manage… but how? Recent developments in the Italian NHS. BMC Health Serv Res. 2016;16(2):65–72. 10.1186/s12913-016-1394-6 PMC4896254 27230750

[10] Fanelli S, Pratici L, Zangrandi A (2022). Managing healthcare services: are professionals ready to play the role of manager?. Health Serv Manage Res.

[11] Day P, Klein R (1983). Two views on the Griffiths report. The mobilisation of consent versus the management of conflict: decoding the Griffiths report. Br Med J.

[12] Flynn R (2004). Remodelling hospitals and health professions in Europe. Social Health Illn.

[13] Skirbekk H, Hem MH, Nortvedt P (2018). Prioritising patient care: the different views of clinicians and managers. Nurs Ethics.

[14] Spyridonidis D, Hendy J, Barlow J (2015). Understanding hybrid roles: the role of identity processes amongst physicians. Public Adm.

[15] Mintzberg H (1973). The nature of managerial work.

[16] Lega F, Rotolo A, Sartirana M (2022). Dealing with pluralism: the managerial work of CEOs in italian public healthcare organizations. BMC Health Serv Res.

[17] Johnson B, Dobni D (2016). Is managerial work in the Public and private sectors really “Different”? A comparative study of managerial work activities. Int J Public Adm.

[18] Porter M, Nohria N (2018). How CEOs manage time. Harvard Business Rev.

[19] Kurke L, Aldrich H (1984). Mintzberg was right: a replication and extension of the nature of managerial work. Manage Sci.

[20] Tengblad S (2006). Is there a ‘new managerial work’? A comparison with Henry Mintzberg’s classic study 30 years later. J Manage Stud.

[21] Bandiera O, Hansen S, Prat A, Sadun R. CEO behavior and form performance. Working Paper 23248, National Bureau of Economic Research. 2017.

[22] Mankins MC (2004). Stop wasting valuable time. Harv Bus Rev.

[23] Braithwaite J, Westbrook MT. (2011). Time spent by health managers in two cultures on work pursuits: real time, ideal time and activities’ importance. Int J Health Plan Manag. 2011; 26(1): 56–69. 10.1002/hpm.1033 22392795

[24] Ruan S, Ma Y. Real-time energy management strategy based on driver-action-impact MPC for series hybrid electric vehicles. Complexity. 2020; 2020:1–15.

[25] Mintzberg H (1998). Managerial work: analysis from observation.

[26] Schaeffer NC, Dykema J, Maynard DW (2010). Interviewers and interviewing. Handb Surv Res.

[27] Fanelli S, Lanza G, Enna C, Zangrandi A (2020). Managerial competences in public organisations: the healthcare professionals’ perspective. BMC Health Serv Res.

[28] Fitzgerald L, Sturt J (1992). Clinicians into management: on the change agenda or not?. Health Serv Manage Res.

[29] Inamdar N, Kaplan RS, Reynolds K (2002). Applying the balanced scorecard in healthcare provider organizations/Practitioner’s response. J Healthc Manag.

[30] Ricciardi W, Tarricone R (2021). The evolution of the Italian National Health Service. Lancet.

[31] Dopson S, Leopold J, Hughes M, Glover I (1996). Doctors in management: a challenge to established debates. Beyond reason? The National Health Service and the limits of management.

[32] Forbes T, Hallier J, Kelly L (2004). Doctors as managers: investors and reluctants in a dual role. Health Serv Manage Res.

[33] Edwards N (2003). Doctors and managers: poor relationships may be damaging patients—what can be done?. BMJ Qual Saf.

[34] Lega F, Prenestini A. (2009). Medico-manager, medico e manager o management del medico. L’aziendalizzazione della sanità in Italia, Rapporto OASI 2009. Milano.

[35] Lalonde C (2007). Crisis management and organizational development: towards the conception of a learning model in crisis management. Organ Dev J.

[36] Carberry JC, Amatoury J, Eckert DJ (2018). Personalized management approach for OSA. Chest.

[37] Smith-Daniels VL, Schweikhart SB, Smith‐Daniels DE (1988). Capacity management in health care services: review and future research directions. Decis Sci.

[38] Mintzberg H (1987). Crafting strategy.

[39] Jones G (2013). Organizational theory, design, and change.

[40] Dopson S, Stewart R (1990). What is happening to middle management?. Br J Manag.

[41] Tello-Leal E, Chiotti O, Villarreal PD (2012). Process-oriented integration and coordination of healthcare services across organizational boundaries. J Med Syst.

[42] Rademakers JJ, De Rooy N, Ten Cate OTJ (2007). Senior medical students’ appraisal of CanMEDS competencies. Med Educ.

[43] Mountford J, Webb C. (2009). When clinicians lead.The McKinsey Quarterly,1.

[44] Glynn RW, Kerin MJ (2010). Factors influencing medical students and junior doctors in choosing a career in surgery. The surgeon.

[45] Arora S, Sevdalis N, Suliman I, Athanasiou T, Kneebone R, Darzi A. (2009). What makes a competent surgeon?: experts’ and trainees’ perceptions of the roles of a surgeon. Am J Surg. 2009;198(5):726–732. 10.1016/j.amjsurg.2009.01.015 19887202

[46] Pavolini E, Vicarelli G (2012). Is decentralization good for your health? Transformations in the italian NHS. Curr Sociol.

[47] Fanelli S, Zangrandi A (2017). Assessment for improving the performance of NICUs: the italian experience. Health Serv Manage Res.

[48] Marcon G, Panozzo F (1998). Reforming the reform: changing roles for accounting and management in the italian health care sector. Eur Acc Rev.

[49] Hofstee WKB, Ten Berge JMF, Hendriks AAJ (1998). How to score questionnaires. Pers Individ Differ.

[50] Ferlie E, Ongaro E (2015). Strategic management in public services organizations: concepts, schools and contemporary issues.

[51] Pine KH, Bossen C, Chen Y, Ellingsen G, Grisot M, Mazmanian M, Møller NH. Data work in healthcare: Challenges for patients, clinicians and administrators. In Companion of the 2018 ACM Conference on Computer Supported Cooperative Work and Social Computing; 2018. p. 433–439.

[52] Oppi C, Vagnoni E (2020). Management accountants’ role and coercive regulations: evidence from the italian health-care sector. Qual Res Account Manag.

[53] Smeltzer LR, Fann GL (1989). Comparison of managerial communication patterns in small, entrepreneurial organizations and large, mature organizations. Group Organ Manag.

[54] Daidone S, D’Amico F (2009). Technical efficiency, specialization and ownership form: evidences from a pooling of italian hospitals. J Prod Anal.

[55] Signorelli C, Odone A, Gozzini A, Petrelli F, Tirani M, Zangrandi A, Zoni R, Florindo N (2017). The missed constitutional reform and its possible impact on the sustainability of the italian national health service. Acta Biomed.

[56] Toth F. (2016). The Italian NHS, the public/private sector mix and the disparities in access to healthcare. Glob Soc Welf. 2016; 3(3):171–178.

[57] Garattini L, Zanetti M, Freemantle N (2020). The italian NHS: what lessons to draw from COVID-19?. Appl Health Econ Health Policy.

[58] Calciolari S, Ilinca S (2016). Unraveling care integration: assessing its dimensions and antecedents in the Italian Health System. Health Policy.

