# Supplementary material for: The managerial role of healthcare professionals in public hospitals: a time-driven analysis of their activities
Source: BMC Health Serv Res. 2023 May 10;23:465. doi: 10.1186/s12913-023-09395-7 (PMC10173533; doi:10.1186/s12913-023-09395-7)
Supplement: Supplementary file 1 — Supplementary Material 1 [file 12913_2023_9395_MOESM1_ESM.docx]

**Appendix 1 – Description of the sample with respondents’ characteristics related to the Questionnaire**

| *Nr. of clinician manager in public Italian hospitals* | | | *6112* |  | *100,00%* |
| --- | --- | --- | --- | --- | --- |
| *Nr. of respondents that accepted to fill in the questionnaire* | | | *2011* |  | *32,90%* |
| *Total nr. of respondents that filled in the questionnaire* | | | *1012* |  | *16,56%* |
| **SAMPLE DESCRIPTION** | | | | | |
|  |  | **Frequency** | **Total** | **Mean/Median** | **%** |
| **Gender of the respondent** | |  | 1.012 |  |  |
|  | ***Male*** | 793 |  |  | 78,36% |
|  | ***Female*** | 212 |  |  | 20,95% |
|  | ***Other/Not specified*** | 7 |  |  | 0,69% |
| **Age of respondents** | |  | 1.012 |  |  |
|  | ***Mean*** |  |  | 60,05 |  |
|  | ***Median*** |  |  | 61,00 |  |
|  | ***Less than 40*** | - |  |  | 0,00% |
|  | ***41-50*** | 54 |  |  | 5,34% |
|  | ***51-60*** | 631 |  |  | 62,35% |
|  | ***More than 60*** | 320 |  |  | 31,62% |
|  | ***Not specified*** | 7 |  |  | 0,69% |
| **Geographical distribution** | |  | 1.012 |  | 61,96% |
|  | ***North*** | 627 |  |  | 19,66% |
|  | ***Center*** | 199 |  |  | 17,69% |
|  | ***South*** | 179 |  |  | 0,69% |
|  | ***Not specified*** | 7 |  |  | 0,00% |
| **Experience as a clinician-manager** | |  | 1.012 |  |  |
|  | ***Mean*** |  | 19 |  |  |
|  | ***Median*** |  | 8 |  |  |
|  | ***Less than 5 years*** | 342 |  |  | 33,79% |
|  | ***5 to 9 years*** | 217 |  |  | 21,44% |
|  | ***10 to 14 years*** | 246 |  |  | 24,31% |
|  | ***15 to 19 yesrs*** | 91 |  |  | 8,99% |
|  | ***20 or more years*** | 112 |  |  | 11,07% |
|  | ***Not specified*** | 4 |  |  | 0,40% |
| **Professional category** | |  | 1.012 |  |  |
|  | ***Medical*** | 295 |  |  | 29,15% |
|  | ***Surgical*** | 226 |  |  | 22,33% |
|  | ***Psychiatric*** | 43 |  |  | 4,25% |
|  | ***Emergency Room*** | 41 |  |  | 4,05% |
|  | ***Territorial health*** | 110 |  |  | 10,87% |
|  | ***Other services*** | 295 |  |  | 29,15% |
|  | ***Not specified*** | 2 |  |  | 0,20% |
| **Size of the unit** | |  | 1.012 |  |  |
|  | ***Mean*** |  |  | 54,12 |  |
|  | ***Median*** |  |  | 28,27 |  |
|  | ***Less than 20 personnel units*** | 159 |  |  | 15,71% |
|  | ***More than 20 personnel units*** | 853 |  |  | 84,29% |
|  | ***Not specified*** | - |  |  | 0,00% |
