# Supplementary material for: The managerial role of healthcare professionals in public hospitals: a time-driven analysis of their activities
Source: BMC Health Serv Res. 2023 May 10;23:465. doi: 10.1186/s12913-023-09395-7 (PMC10173533; doi:10.1186/s12913-023-09395-7)
Supplement: Supplementary file 2 — Supplementary Material 2 [file 12913_2023_9395_MOESM2_ESM.docx]

**Appendix 2 – Questionnaire on time dedicated to management activities**

The present questionnaire is administered online to healthcare professionals holding a middle management position in order to identify how much time do they spent on management activities and how they feel it adequate.

**BODY OF THE QUESTIONNAIRE**

**SECTION A – PERSONAL INFORMATION**

1. Gender

- Male
- Female
- Other
- Prefer not to say

1. Age: _____
2. Region in which you work: _____
3. Type of hospital in which you work:

- Public
- Private

1. How many years have you been managing your unit? ____
2. How many medical employees has your unit? _____

**SECTION B – QUESTIONS**

1. If 100 is your total professional time, how much time do you spend on each of the following activities?

| Activity | Time spent (min 0 max 100) |
| --- | --- |
| Clinical activity (e.g., patients care, clinical records, patients’ relations, etc.) |  |
| Management activities (e.g., auditing, definition of care pathways, guidelines, personnel evaluation, etc.) |  |
| Non-clinical activity (e.g., emails, formal procedures, shift planning, etc.) |  |
| Internal relations within the organisation (e.g., formal or informal meetings, relations with colleagues fellows’ relations, etc.) |  |
| External relations with stakeholders (e.g., budgeting, trade union relations, committees, providers, other organizations, etc.) |  |
| Education and research activities |  |

1. *How do you assess the time you spend on each activity? (1 = insufficient; 3 = adequate; 5 = excessive)*

|  | 1 = insufficient | 2 | 3 = adequate | 4 | 5 = excessive |
| --- | --- | --- | --- | --- | --- |
| Clinical activity (e.g., patients care, clinical records, patients’ relations, etc.) |  |  |  |  |  |
| Management activities (e.g., auditing, definition of care pathways, guidelines, personnel evaluation, etc.) |  |  |  |  |  |
| Non-clinical activity (e.g., emails, formal procedures, shift planning, etc.) |  |  |  |  |  |
| Internal relations within the organisation (e.g., formal or informal meetings, relations with colleagues fellows’ relations, etc.) |  |  |  |  |  |
| External relations with stakeholders (e.g., budgeting, trade union relations, committees, providers, other organizations, etc.) |  |  |  |  |  |
| Education and research activities |  |  |  |  |  |
